# Ovarian germline stem cells

**DOI:** 10.1186/scrt487

**Published:** 2014-08-18

**Authors:** Cheryl E Dunlop, Evelyn E Telfer, Richard A Anderson

**Affiliations:** MRC Centre for Reproductive Health, Queen’s Medical Research Institute, The University of Edinburgh, 47 Little France Crescent, Edinburgh, EH16 4TJ UK; Institute of Cell Biology and Centre for Integrative Physiology, Hugh Robson Building, University of Edinburgh, George Square, Edinburgh EH8 9XD UK

## Abstract

It has long been established that germline stem cells (GSCs) are responsible for lifelong gametogenesis in males, and some female invertebrates (for example, *Drosophila*) and lower vertebrates (for example, teleost fish and some prosimians) also appear to rely on GSCs to replenish their oocyte reserve in adulthood. However, the presence of such cells in the majority of female mammals is controversial, and the idea of a fixed ovarian reserve determined at birth is the prevailing belief among reproductive biologists. However, accumulating evidence demonstrates the isolation and culture of putative GSCs from the ovaries of adult mice and humans. Live offspring have been reportedly produced from the culture of adult mouse GSCs, and human GSCs formed primordial follicles using a mouse xenograft model. If GSCs were present in adult female ovaries, it could be postulated that the occurrence of menopause is not due to the exhaustion of a fixed supply of oocytes but instead is a result of GSC and somatic cell aging. Alternatively, they may be benign under normal physiological conditions. If their existence were confirmed, female GSCs could have many potential applications in both basic science and clinical therapies. GSCs not only may provide a valuable model for germ cell development and maturation but may have a role in the field of fertility preservation, with women potentially being able to store GSCs or GSC-derived oocytes from their own ovaries prior to infertility-inducing treatments. Essential future work in this field will include further independent corroboration of the existence of GSCs in female mammals and the demonstration of the production of mature competent oocytes from GSCs cultured entirely *in vitro*.

## Introduction

Germline stem cells (GSCs) are a unique cell population committed to producing gametes for the propagation of the species. The concept of a GSC most likely originates from Regaud [1, 2], whose work on spermatogenesis was published over a century ago. He postulated that, in order for sperm production to occur, a population of self-renewing cells must be present in the testis which could produce differentiated progeny. It is now well established that these cells, now known as spermatogonial stem cells, contribute to spermatogenesis in adulthood in the males of all species studied [3]. Research on the existence of a female counterpart, an ovarian GSC that is able to undergo postnatal *neo*-oogenesis and thus contribute to oocyte production in adulthood, has revealed a more complicated picture. Although female GSCs (fGSCs) appear to have a role in oogenesis throughout reproductive life in some non-mammalian species, these examples appear to be relatively rare across the phyla of the animal kingdom [4], and the presence of fGSCs in mammals has been greatly debated. Indeed, the prevailing view is that female mammals are born with a finite stock of mature oocytes that become exhausted with aging, a hypothesis first suggested by the 19th century embryologist Waldeyer [5]. General opinion changed at the beginning of the 20th century when the prevailing belief was in favor of *neo*-oogenesis in adulthood [6] until an influential article by Zuckerman [7] in 1951 reported no evidence that new oocytes are formed once a female is born, and the idea of a fixed ovarian reserve in mammals has been a central dogma in the field since. However, since 2004, a growing number of researchers have found cause to question this doctrine. The debate was reignited with the proposition [8], and subsequent isolation [9–13], of purported fGSCs (also known as oogonial stem cells, or OSCs).

Critically, the physiological role of these cells *in vivo* in the adult mammalian ovary has yet to be determined. Development and maturation of an oocyte entail a complex and multifaceted process which has to be tightly regulated in order for the oocyte to be competent for fertilization. This includes bidirectional communication between the oocyte and its surrounding somatic cells, precise timing of cessation and resumption of meiosis, and correct genomic imprinting (reviewed last year by Li and Albertini [14] and Anckaert and colleagues [15]). Imprinting involves epigenetic alterations of the parental alleles by DNA methylation and determines whether the maternal or paternal gene will be expressed in the embryo. Incorrect imprinting can lead to conditions such as Angelman and Prader-Willi syndromes. Therefore, future research involving the culture of oocytes derived from purported adult mammalian fGSCs will have to ensure that these processes are intact for these cells to be useful in clinical practice. This review will examine the existence of OSCs in various species, consider where research in the field is heading, and assess the therapeutic potential of such cells.

### Ovarian germline stem cells in non-mammalian species and prosimian primates

There are several animals in which fGSCs actively replenish the ovarian reserve postnatally. fGSCs in ‘lower’ invertebrates have been extensively studied in the fruit fly, *Drosophila* [4]. In this species, a few primordial germ cells (PGCs) are effectively ‘segregated’ in a special germ cell niche at the tip of each ovariole (16 to 18 tubes that make up the ovary) prenatally [16]. The environment within this niche, in contrast to environments elsewhere in the ovary, prevents the PGCs from differentiating, and these undifferentiated cells subsequently become fGSCs [17]. Postnatally, this niche controls the division of fGSCs and the production of new oocytes, therefore providing a continuous supply of germ cells throughout reproductive life.

fGSCs have also been reported in teleost fish, including the medaka (*Oryzias latipes*) [18] and zebrafish (*Danio rerio*) [19]. As in *Drosophila*, medaka have a germ cell niche, called the germinal cradle, situated in the ovarian cords [18]. Within this area reside mitotic cells that have the characteristics of fGSCs and that continuously supply the ovary with new oocytes. Furthermore, zebrafish possess a distinct zone on the ovarian surface to which germ cells are confined, and this may also be analogous to the *Drosophila* germ cell niche [19], suggesting evolutionary conservation across animal phyla. Oogenesis throughout reproductive life may be necessary for the huge numbers of eggs produced during the fish and fly life span and appears more similar to spermatogenesis than the restrictive processes of oogenesis and associated follicle development in higher mammals.

Although prior to 2004 it was widely believed that the vast majority of adult mammals lack fGSCs, a few exceptions had been described. The adult ovaries of two members of the loris family, which are prosimians related to the lemur, have been reported to possess mitotically active germ cells located within ‘nests’ in the ovarian cortex [20–22]. It has not been proven, however, that these cells, found in a slow loris (*Nycticebus coucang*) and a slender loris (*Loris tardigradus lydekkerianus*), are actually capable of undergoing folliculogenesis and producing mature oocytes.

If fGSCs can be identified in such animals, why would they not be present in the ovaries of the vast majority of adult female mammals? Zuckerman himself was actually an advocate for *neo*-oogenesis until his convictions were changed by his extensive review of the literature [23], in which he stated: ‘None of the experimental and quantitative evidence which we have considered thus supports the view that oogenesis occurs in the adult ovary, and much of it bears very clearly against the proposition’ [7].

Lack of evidence is not definitive, and proving that a cell does not exist is difficult, especially if they are a scarce population. So what is the evidence for the existence of fGSCs in adult mammals?

### Ovarian germline stem cells in mammals

The discovery of purported fGSCs in adult mice occurred during an investigation of oocyte atresia and its role in follicular dynamics, when an apparent mathematical anomaly was observed. Johnson and colleagues [8] reported that follicular atresia was occurring at a rate such that the adult mouse should exhaust her ovarian reserve well before the age that it in fact occurs. This implied that the follicle pool must be replenished in adulthood by *neo*-oogenesis in order to sustain the reproductive life of the mouse, and considering the germ cell dynamics model of Faddy and colleagues [24], the authors suggested that the adult mouse has to make 77 new primordial follicles a day. On further investigation, a rare population of mitotically active ovoid cells in the ovarian surface epithelium (OSE), which expressed the germ cell-specific protein mouse vasa homolog (MVH), was identified. Furthermore, when small pieces of wild-type ovarian cortex were transplanted onto the ovaries of transgenic mice that ubiquitously expressed green fluorescent protein (GFP) for 3 to 4 weeks, GFP-positive oocytes surrounded by wild-type somatic cells were found within the wild-type graft. These results persuaded the authors that new oocytes must continue to be produced throughout reproductive life in mice and that the proliferating cells in the OSE may be putative fGSCs and therefore the source of the ongoing oogenesis.

The article by Johnson and colleagues was met with widespread criticism, and subsequent work from the Tilly group, who suggested that the source of these fGSCs was the bone marrow and peripheral blood [25], was even more controversial [26, 27]. However, Zou and colleagues [9] took a step forward when they reported the isolation of fGSCs from adult mice. Using a magnetically activated cell sorting technique, the authors isolated putative fGSCs measuring 12 to 20 μm in diameter by using an antibody against either DDX4 (DEAD box polypeptide 4; also known as vasa or MVH) or IFITM3 (interferon-induced transmembrane protein 3; also known as fragilis) [9, 11]. These cells expressed both pluripotency and germ cell markers, had a normal karyotype, and were maternally imprinted. Evidence of their capacity to undergo oogenesis was provided when GFP-expressing fGSCs were transplanted into sterilized mice, with GFP-positive offspring being produced. These findings in adult mice were supported by subsequent articles by Pacchiarotti and colleagues [10] and Hu and colleagues [12], who reported isolation of putative fGSCs by using different techniques, though with limited demonstration of oocyte-like competence. The first, and only, published evidence of the existence of these cells in humans was provided by the Tilly group in 2012 [13]. White and colleagues [13] developed a fluorescence-activated cell sorting protocol that consistently isolated fGSCs, which the authors named OSCs, from both adult mice and humans. Measuring 5 to 8 μm, the cells were smaller than those isolated by Zou and colleagues [9] but expressed similar germ cell markers. The reason these cells have not been detected in the past may be explained by the fact that White and colleagues [13] estimated that the OSC population makes up only 0.014 % ± 0.002 % of the mouse ovary. The authors noted spontaneous production of oocyte-like cells from fGSCs in *in vitro* culture (also observed by Pacchiarotti and colleagues [10]); these cells showed expression of oocyte-specific and meiotic markers. Finally, by injecting GFP-expressing fGSCs into non-GFP ovarian cortex and xenotransplanting the tissue into mice, the authors reported that primordial follicles comprising a GFP-positive oocyte and wild-type granulosa cells could be seen on removal of the graft.

In addition to these putative fGSCs, another population of ovarian stem cells that reportedly differentiate into oocytes has been isolated from the OSE [28–30]. These cells, named very small embryonic-like (VSEL) stem cells, are cultured from OSE scrapings, are smaller than the fGSCs discussed above, and differ in morphology from those reported by White and colleagues [13]. The cells express a number of stem cell markers, including SSEA-4, and spontaneously generate large, oocyte-like cells in culture. Interestingly, Parte and colleagues [29] also isolated a second putative ovarian stem cell population, slightly larger in size than the VSEL stem cells and perhaps more analogous to fGSCs. They postulated that the VSEL stem cells are, in fact, the precursors of these larger cells, which may be tissue-committed ovarian stem cells [29]. To date, VSEL stem cells have been reported in adult mice, rabbits, sheep, marmoset monkeys, and humans [28, 29], including postmenopausal women and women with premature ovarian insufficiency [31]. VSEL stem cells from the OSE would appear to be distinct from fGSCs; however, the existence of VSEL stem cells, much like that of fGSCs, has also been controversial [32].

More recent evidence for the existence of mammalian fGSCs has been published by a Mexican group working with three species of phyllostomid bats [33]. The use of these species of bats is especially pertinent because they share some reproductive similarities with primates, both anatomically and with respect to ovulation patterns. For example, *Glossophaga soricina* are polyoestrous mono-ovulates with menstrual cycles of 22 to 26 days, including a luteal phase and periodic endometrial shedding [34]. Antonio-Rubio and colleagues [33] demonstrated that the ovaries of *Artibeus jamaicensis*, *Glossophaga soricine*, and *Sturnira lilium* are polarized, with a medullary region containing developing follicles and a cortical region containing both primordial follicles and a population of cells which looked similar to germ cells histologically. These cells, when analyzed with immunofluorescence, expressed proliferation, pluripotency, and early germline markers, including phosphorylated histone H3, POU5F1, DDX4, and IFITM3, and were termed adult cortical germ cells (ACGCs). The authors thus postulated that ACGCs may be involved in adult *neo*-oogenesis in these species, although, as with the loris species mentioned previously, this was not demonstrated in this study.

In addition to this emergent body of proof, there is indirect evidence to support *neo*-oogenesis in adult female mammals. Work on rhesus monkey ovaries in the 1950s demonstrated findings similar to those of Johnson and colleagues [8] in the mouse, with the observed rates of follicular atresia predicting that monkey ovarian reserve should be depleted within 2 years [35]. The author calculated that the maximum life span of an oocyte was 2 years, and therefore the data suggested that the new oocytes must be continually produced throughout reproductive life. Mathematical modeling has provided conflicting data, and both Bristol-Gould and colleagues [36] and Wallace and Kelsey [37] found that the ‘germline stem cell model’ did not fit follicular kinetics data in either mice [36] or humans [37]. Conversely, Kerr and colleagues [38] have published data in support of postnatal oogenesis. Although they did not find evidence of GSCs, the authors demonstrated that the mean number of primordial follicles in mice did not decline between days 7 and 100 of age, leading them to surmise that there is a mechanism by which postnatal neo-folliculogenesis sustains the follicular pool.

Further indirect evidence has come from lineage tracing, although this has also provided conflicting evidence; some data have refuted the fGSC hypothesis, and some have been unable to disprove that postnatal neo-oogenesis exists [39, 40]. Lei and Spradling [39] have reported that primordial follicles are very stable, with no evidence of high rates of turnover, therefore suggesting that the pool is sufficient to sustain fertility without the requirement of fGSCs. In contrast, by examining the accumulation of microsatellite mutations in mice, Reizel and colleagues [40] found that oocyte ‘depth’ increased with age; in other words, the older the mouse, the more mitotic divisions the oocyte has undergone. If neo-oogenesis were not occurring postnatally, then depth should be stable throughout life and independent of any interventions; however, depth was also shown to increase after ovariectomy and this would indicate neo-oogenesis [41]. The ‘production-line hypothesis’ of Henderson and Edwards [42] may go some way to explaining this observation; however, the formation of new oocytes after birth is a possible alternative explanation. The findings of Lei and Spradling have also been refuted by Bhartiya and colleagues [43], who observed germ cell ‘cysts’ in adult mice and sheep which, the authors believe, reflect clonal expansion of stem cells within the ovary.

### Germline stem cell aging

If ovaries *are* capable of producing new oocytes during adulthood, then the obvious question is: why do women go through menopause? It has traditionally been believed that women enter menopause when their finite supply of oocytes has been exhausted; however, if *neo*-oogenesis does indeed exist, then the rate of new oocyte production must lessen with age in order for menopause to occur. There may be two potential underlying mechanisms: failure of the fGSCs to form oocytes or failure of the somatic environment to support oocyte development (or both). It is possible that fGSCs, like many other cells, undergo an aging process and thus lose their capacity to regenerate and differentiate. In *Drosophila*, there is a reduction in oocyte production with age, associated with declining rates of fGSC division and increased apoptosis of developing oocytes [44]. Furthermore, an age-dependent deterioration in germ cell niche signaling may affect the ability of fGSCs to regenerate [44]. Several putative causative mechanisms for these age-related changes have been proposed, including a decrease in bone morphogenetic protein (BMP) production by the germ cell niche, a reduction in GSC-niche cell adhesion via E-cadherin, and an increase in harmful reactive oxygen species [45]. Manipulation of all of these factors has been shown to increase fGSC life span [45].

It is likely that mammalian aging can also be attributed, at least partly, to age-related stem cell senescence, and hematopoietic, neural, and muscle stem cell function all demonstrate a decline in function (reviewed in [46]). With regard to ovarian GSCs, Pacchiarotti and colleagues [10] found that the number of fGSCs they were able to isolate from mouse ovaries diminished with increasing age of the mouse. Furthermore, a study has reported the presence of putative fGSCs in aged mice that appear to undergo folliculogenesis only when transplanted back into a young mouse ovary, thus implying that the surrounding ovarian environment may have a role to play in the ability of fGSCs to sustain a woman’s reproductive function [47]. Therefore, the idea that the existence of menopause renders *neo*-oogenesis impossible is not necessarily correct: the two phenomena may co-exist. However, the key demonstration that fGSCs contribute to the postnatal follicle pool and potentially to fertility in a physiological context has not been made. Although fGSCs may be isolatable from ovarian tissue and potentially able to form oocytes within follicles after various manipulations, this may occur only under experimental conditions and they may not have any relevance to the normal processes of ovarian function.

### Basic science uses for germline stem cells

The potential uses for fGSCs are numerous, particularly in basic science but potentially even in clinical applications. With regard to the former, fGSCs provide an exciting prospect as a germ cell model in order to study the development and maturation of the oocyte. Park and colleagues [48] have used adult mouse-derived fGSCs to investigate the effect of BMP4. BMPs are a member of the transforming growth factor-beta family of growth factors with a critical role in PGC specification [49, 50] and have been shown to act on germ cells within the developing human ovary [51]. Treatment of fGSCs with BMP4 increased both the rate of *in vitro* differentiation into oocyte-like structures and the expression of genes associated with the initiation of meiosis: muscle-segment homeobox 1 (Msx1), Msx2, and stimulated by retinoic acid gene 8 (Stra8) [48].

fGSCs have also been genetically manipulated to produce transgenic mice. Zhang and colleagues [52] transfected female adult mouse GSCs with recombinant viruses containing vectors for different genes, including GFP. When transplanted into sterilized mice and mated with wild-type male mice, offspring heterozygous for the transfected genes were produced. Using a liposome-mediated transfection, the same group was also able to create a knockout mouse to investigate the role of the gene *Oocyte*-*G1* [52]. The ability to produce transgenic animals in this way could be an excellent tool for reproductive biologists in the future.

### Therapeutic uses for germline stem cells

There is no doubt that if fGSCs can be shown to develop into mature, competent, correctly imprinted oocytes *in vitro*, they will have great clinical potential; however, owing to technical and regulatory issues, it may be a long time before this potential can be fulfilled. For example, in the UK, research into whether fGSC-derived oocytes would be capable of fertilization and development into a blastocyst would be possible only with the approval of the Human Fertilization and Embryology Authority. Nevertheless, fGSCs *may* have a role in both fertility preservation and the reversal of reproductive senescence. With regard the former, it is conceivable that fGSCs could be used as a fertility preservation strategy for women who require gonadotoxic treatment for cancer that may render them infertile. A sample of ovarian cortex could be taken prior to commencing treatment, and fGSCs could be isolated and cryopreserved for future use. The fGSCs, when required, could subsequently be injected back into a woman’s ovaries where they could undergo *neo*-folliculogenesis, or they could be cultured *in vitro* in ovarian cortex to a mature oocyte stage and resultant oocytes used in *in vitro* fertilization (IVF). The benefits of this approach are twofold: firstly, taking ovarian cortex samples would not require life-saving treatment to be delayed in contrast to the ovarian superovulation regimens required for oocyte and embryo cryopreservation; secondly, many more new follicles and oocytes could be achieved from fGSCs than would be present in cryopreserved tissue or from ovarian stimulation.

Women with age-related infertility or premature ovarian insufficiency may also benefit from fGSCs. ‘Social’ oocyte storage is becoming increasingly sought by women who are anxious about how much longer their ovarian reserve will last. However, this is an expensive endeavor, is not without health risks, and may result in only a small number of oocytes being cryopreserved. As mentioned previously, putative fGSCs have been reported in aged mice [47]; therefore, it is not impossible that women who are perimenopausal, prematurely or not, may have a very small number of these cells residing in their ovaries. The prospect of these cells growing into oocytes in the aged stromal environment is less certain; however, they may be able to be used in IVF. The idea of ‘reversing’ the reproductive clock and thereby avoiding the detrimental health effects and climacteric symptoms of menopause is appealing to some; however, the aging ovarian milieu may also restrict the use of fGSCs to this end. In summary, such clinical applications are currently aspirational but worthy of further investigation.

### Germline stem cells – the future

The field of reproductive biology remains very skeptical of the idea that female mammalian GSCs exist and particularly that they have any physiological role in normal ovarian function. Further demonstration of their isolation and *in vitro* characteristics from a range of species is needed as a first step. The potential for fGSCs to differentiate into daughter cells that become mature oocytes in an *in vivo* environment remains to be demonstrated. Given the apparent scarcity of fGSCs in the female mouse ovary, this may prove difficult to demonstrate. For those groups who have already isolated putative fGSCs, the essential next steps are investigating the conditions under which these cells will develop into oocytes that are capable of fertilization and thus exploring their potential as gametes. For fGSCs to be used in a clinical context, a complete *in vitro* culture system will need to be developed. In this regard, we are currently investigating whether fGSCs can be grown into a mature oocyte by using a multi-step serum-free culture system that we have already shown promotes healthy follicular growth in bovine and human ovarian cortex [53–55].

## Conclusions

The reported existence of female mammalian GSCs has stimulated much interest among reproductive biologists, many of whom are yet to be convinced that these cells are a real entity. However, there are now a growing number of reports of their isolation and culture, and strides are being taken to investigate their *neo*-oogenesis capabilities. Whether these cells have a physiological role has yet to be determined, and concerns remain that isolated putative fGSCs have undergone *in vitro* transformation in order to form oocytes; yet if their potential can be harnessed, they may contribute greatly to our understanding of oocyte development and may be of important clinical relevance.

## Abbreviations

ACGC: Adult cortical germ cell
BMP: Bone morphogenetic protein
DDX4: DEAD box polypeptide 4
fGSC: Female germline stem cell
GFP: Green fluorescent protein
GSC: Germline stem cell
IFITM3: Interferon-induced transmembrane protein 3
IVF: *in vitro* fertilization
MSX: Muscle-segment homeobox
MVH: Mouse vasa homolog
OSC: Oogonial stem cell
OSE: Ovarian surface epithelium
PGC: Primordial germ cell
VSEL: Very small embryonic-like.

## References

[1] Regaud C (1901). Etudes sur la structure des tubes seminiferes et sur la spermatogenese chez les mammiferes [Studies on the structure of seminiferous tubules and on spermatogenesis in mammals]. Part 1. Archives d’Anatomie microscopiques et de Morphologie experimentale.

[2] Regaud C (1901). Etudes sur la structure des tubes seminiferes et sur la spermatogenese chez les mammiferes [Studies on the structure of seminiferous tubules and on spermatogenesis in mammals] . Part 2. Archives d’Anatomie microscopiques et de Morphologie experimentale.

[3] Brinster RL (2007). Male germline stem cells: from mice to men. Science.

[4] Spradling A, Fuller MT, Braun RE, Yoshida S (2011). Germline stem cells. Cold Spring Harb Perspect Biol.

[5] Waldeyer W (1870). Eierstock und Ei [Ovary and egg].

[6] Allen E (1923). Ovogenesis during sexual maturity. Am J Anat.

[7] Zuckerman S (1951). The number of oocytes in the mature ovary. Rec Prog Horm Res.

[8] Johnson J, Canning J, Kaneko T, Pru JK, Tilly JL (2004). Germline stem cells and follicular renewal in the postnatal mammalian ovary. Nature.

[9] Zou K, Yuan Z, Yang Z, Luo H, Sun K, Zhou L, Xiang J, Shi L, Yu Q, Zhang Y, Hou R, Wu J (2009). Production of offspring from a germline stem cell line derived from neonatal ovaries. Nat Cell Biol.

[10] Pacchiarotti J, Maki C, Ramos T, Marh J, Howerton K, Wong J, Pham J, Anorve S, Chow YC, Izadyar F (2010). Differentiation potential of germ line stem cells derived from the postnatal mouse ovary. Differentiation.

[11] Zou K, Hou L, Sun K, Xie W, Wu J (2011). Improved efficiency of female germline stem cell purification using fragilis-based magnetic bead sorting. Stem Cells Dev.

[12] Hu Y, Bai Y, Chu Z, Wang J, Wang L, Yu M, Lian Z, Hua J (2012). GSK3 inhibitor-BIO regulates proliferation of female germline stem cells from the postnatal mouse ovary. Cell Prolif.

[13] White YA, Woods DC, Takai Y, Ishihara O, Seki H, Tilly JL (2012). Oocyte formation by mitotically active germ cells purified from ovaries of reproductive-age women. Nat Med.

[14] Li R, Albertini DF (2013). The road to maturation: somatic cell interaction and self-organization of the mammalian oocyte. Nat Rev Mol Cell Biol.

[15] Anckaert E, De Rycke M, Smitz J (2013). Culture of oocytes and risk of imprinting defects. Hum Reprod Update.

[16] Xie T, Spradling AC (2000). A niche maintaining germ line stem cells in the Drosophila ovary. Science.

[17] Gilboa L, Lehmann R (2004). Repression of primordial germ cell differentiation parallels germ line stem cell maintenance. Curr Biol.

[18] Nakamura S, Kobayashi K, Nishimura T, Higashijima S, Tanaka M (2010). Identification of germline stem cells in the ovary of the teleost medaka. Science.

[19] Draper BW, McCallum CM, Moens CB (2007). nanos1 is required to maintain oocyte production in adult zebrafish. Dev Biol.

[20] David GF, Anand Kumar TC, Baker TG (1974). Uptake of tritiated-thymidine by primordial germinal cells in ovaries of adult slender loris. J Reprod Fertil.

[21] Duke KL (1967). Ovogenetic activity of fetal-type in ovary of adult slow loris Nycticebus coucang. Folia Primatol.

[22] Telfer EE (2004). Germline stem cells in the postnatal mammalian ovary: a phenomenon of prosimian primates and mice?. Reprod Biol Endocrinol.

[23] Zuckerman S (1971). Beyond the Ivory Tower: The Frontiers of Public and Private Science.

[24] Faddy MJ, Telfer E, Gosden RG (1987). The kinetics of pre-antral follicle development in ovaries of CBA/Ca mice during the first 14 weeks of life. Cell Tissue Kinet.

[25] Johnson J, Bagley J, Skaznik-Wikiel M, Lee HJ, Adams GB, Niikura Y, Tschudy KS, Tilly JC, Cortes ML, Forkert R, Spitzer T, Iacomini J, Scadden DT, Tilly JL (2005). Oocyte generation in adult mammalian ovaries by putative germ cells in bone marrow and peripheral blood. Cell.

[26] Telfer EE, Gosden RG, Byskov AG, Spears N, Albertini D, Andersen CY, Anderson R, Braw-Tal R, Clarke H, Gougeon A, McLaughlin E, McLaren A, McNatty K, Schatten G, Silber S, Tsafriri A (2005). On regenerating the ovary and generating controversy. Cell.

[27] Byskov AG, Faddy MJ, Lemmen JG, Andersen CY (2005). Eggs forever?. Differentiation.

[28] Bukovsky A, Svetlikova M, Caudle MR (2005). Oogenesis in cultures derived from adult human ovaries. Reprod Biol Endocrinol.

[29] Parte S, Bhartiya D, Telang J, Daithankar V, Salvi V, Zaveri K, Hinduja I (2011). Detection, characterization, and spontaneous differentiation in vitro of very small embryonic-like putative stem cells in adult mammalian ovary. Stem Cells Dev.

[30] Virant-Klun I, Skutella T, Hren M, Gruden K, Cvjeticanin B, Vogler A, Sinkovec J (2013). Isolation of small SSEA-4-positive putative stem cells from the ovarian surface epithelium of adult human ovaries by two different methods. Biomed Res Int.

[31] Virant-Klun I, Zech N, Rozman P, Vogler A, Cvjeticanin B, Klemenc P, Malicev E, Meden-Vrtovec H (2008). Putative stem cells with an embryonic character isolated from the ovarian surface epithelium of women with no naturally present follicles and oocytes. Differentiation.

[32] Abbott A (2013). Doubt cast over tiny stem cells. Nature.

[33] Antonio-Rubio NR, Porras-Gomez TJ, Moreno-Mendoza N (2013). Identification of cortical germ cells in adult ovaries from three phyllostomid bats: Artibeus jamaicensis, Glossophaga soricina and Sturnira lilium. Reprod Fertil Dev.

[34] Rasweiler JJ (1972). Reproduction in the long-tongued bat, Glossophaga soricina. I. Preimplantation development and histology of the oviduct. J Reprod Fertil.

[35] Vermande-Van Eck GJ (1956). Neo-ovogenesis in the adult monkey - consequences of atresia of ovocytes. Anat Rec.

[36] Bristol-Gould SK, Kreeger PK, Selkirk CG, Kilen SM, Mayo KE, Shea LD, Woodruff TK (2006). Fate of the initial follicle pool: empirical and mathematical evidence supporting its sufficiency for adult fertility. Dev Biol.

[37] Wallace WH, Kelsey TW (2010). Human ovarian reserve from conception to the menopause. PloS One.

[38] Kerr JB, Duckett R, Myers M, Britt KL, Mladenovska T, Findlay JK (2006). Quantification of healthy follicles in the neonatal and adult mouse ovary: evidence for maintenance of primordial follicle supply. Reproduction.

[39] Lei L, Spradling AC (2013). Female mice lack adult germ-line stem cells but sustain oogenesis using stable primordial follicles. Proc Natl Acad Sci U S A.

[40] Reizel Y, Itzkovitz S, Adar R, Elbaz J, Jinich A, Chapal-Ilani N, Maruvka YE, Nevo N, Marx Z, Horovitz I, Wasserstrom A, Mayo A, Shur I, Benayahu D, Skorecki K, Segal E, Dekel N, Shapiro E (2012). Cell lineage analysis of the mammalian female germline. PLoS Genet.

[41] Woods DC, Telfer EE, Tilly JL (2012). Oocyte family trees: old branches or new stems?. PLoS Genet.

[42] Henderson SA, Edwards RG (1968). Chiasma frequency and maternal age in mammals. Nature.

[43] Bhartiya D, Sriraman K, Parte S, Patel H (2013). Ovarian stem cells: absence of evidence is not evidence of absence. J Ovarian Res.

[44] Zhao R, Xuan Y, Li X, Xi R (2008). Age-related changes of germline stem cell activity, niche signaling activity and egg production in Drosophila. Aging Cell.

[45] Pan L, Chen SY, Weng CJ, Call G, Zhu DX, Tang H, Zhang N, Xie T (2007). Stem cell aging is controlled both intrinsically and extrinsically in the Drosophila ovary. Cell Stem Cell.

[46] Signer RA, Morrison SJ (2013). Mechanisms that regulate stem cell aging and life span. Cell Stem Cell.

[47] Niikura Y, Niikura T, Tilly JL (2009). Aged mouse ovaries possess rare premeiotic germ cells that can generate oocytes following transplantation into a young host environment. Aging (Alb any NY).

[48] Park ES, Woods DC, Tilly JL (2013). Bone morphogenetic protein 4 promotes mammalian oogonial stem cell differentiation via Smad1/5/8 signaling. Fertil Steril.

[49] Lawson KA, Dunn NR, Roelen BA, Zeinstra LM, Davis AM, Wright CV, Korving JP, Hogan BL (1999). Bmp4 is required for the generation of primordial germ cells in the mouse embryo. Genes Dev.

[50] Ying Y, Liu XM, Marble A, Lawson KA, Zhao GQ (2000). Requirement of Bmp8b for the generation of primordial germ cells in the mouse. Mol Endocrinol.

[51] Childs AJ, Kinnell HL, Collins CS, Hogg K, Bayne RA, Green SJ, McNeilly AS, Anderson RA (2010). BMP signaling in the human fetal ovary is developmentally regulated and promotes primordial germ cell apoptosis. Stem Cells.

[52] Zhang Y, Yang Z, Yang Y, Wang S, Shi L, Xie W, Sun K, Zou K, Wang L, Xiong J, Xiang J, Wu J (2011). Production of transgenic mice by random recombination of targeted genes in female germline stem cells. J Mol Cell Biol.

[53] Telfer EE, McLaughlin M, Ding C, Thong KJ (2008). A two-step serum-free culture system supports development of human oocytes from primordial follicles in the presence of activin. Hum Reprod.

[54] McLaughlin M, Telfer EE (2010). Oocyte development in bovine primordial follicles is promoted by activin and FSH within a two-step serum-free culture system. Reproduction.

[55] Anderson RA, McLaughlin M, Woods DC, Tilly JL, Telfer EE (2013). Evaluation of oogonial stem cells and neo-oogenesis in ovaries of girls and women with Turner syndrome: a pilot study. Hum Reprod.

